# In Vitro Glucuronidation and Sulfation of ε-Viniferin, a Resveratrol Dimer, in Humans and Rats

**DOI:** 10.3390/molecules22050733

**Published:** 2017-05-03

**Authors:** Arnaud Courtois, Michael Jourdes, Adeline Dupin, Caroline Lapèze, Elodie Renouf, Benoît Biais, Pierre-Louis Teissedre, Jean-Michel Mérillon, Tristan Richard, Stéphanie Krisa

**Affiliations:** 1 Unité de Recherche Œnologie, Molécules d’Intérêt Biologique, EA 4577, USC 1366 INRA, Bordeaux INP, Institut des Sciences de la Vigne et du Vin, 210 Chemin de Leysottes, 33882 Villenave d’Ornon, France; arnaud.courtois@u-bordeaux.fr (A.C.); michael.jourdes@u-bordeaux.fr (M.J.); adeline.dupin@etu.u-bordeaux.fr (A.D.); carolinelapeze@live.fr (C.L.); elodie.renouf@yahoo.fr (E.R.); benoit.biais@u-bordeaux.fr (B.B.); p.teissedre@u-bordeaux2.fr (P.-L.T.); jean-michel.merillon@u-bordeaux.fr (J.-M.M.); tristan.richard@u-bordeaux.fr (T.R.); 2 Université de Bordeaux, 146, rue Léo Saignat, 33076 Bordeaux, France; 3 Centre Antipoison et de Toxicovigilance d’Aquitaine Poitou-Charentes, Bâtiment UNDR, CHU de Bordeaux, Place Amélie Raba Léon, 33076 Bordeaux, France; 4 Polyphénols Biotech, Université de Bordeaux, Institut des Sciences de la Vigne et du Vin, 210 Chemin de Leysottes, 33882 Villenave d’Ornon, France

**Keywords:** ε-viniferin, metabolism, human, rat, liver, sulfation, glucuronidation, hemi-synthesis

## Abstract

ε-Viniferin is a resveratrol dimer that possesses antioxidant or anti-inflammatory activities. However little is known about the metabolism of this oligostilbene. This study was thus undertaken as a first approach to identify and characterize the metabolites of ε-viniferin and to describe the kinetic profile of their appearance in humans and rats. The glucuronides and sulfates of ε-viniferin were first obtained by chemical hemi-synthesis and were fully characterized by UPLC-MS and NMR spectroscopy. Then, ε-viniferin was incubated with human or rat S9 liver fractions that led to the formation of four glucuronoconjugates and four sulfoconjugates. In both species, ε-viniferin was subjected to an intense metabolism as 70 to 80% of the molecule was converted to glucuronides and sulfates. In humans, the hepatic clearance of ε-viniferin (V_max_/K_m_) for glucuronidation and sulfation were 4.98 and 6.35 µL/min/mg protein, respectively, whereas, in rats, the hepatic clearance for glucuronidation was 20.08 vs. 2.59 µL/min/mg protein for sulfation. In humans, three major metabolites were observed: two glucuronides and one sulfate. By contrast, only one major glucuronide was observed in rats. This strong hepatic clearance of ε-viniferin in human and rat could explain its poor bioavailability and could help to characterize its active metabolites.

## 1. Introduction

Stilbenes are naturally-occurring phenolic compounds mainly found in grapes and wine. One of them, resveratrol, has been widely studied. It has been reported to act preventively against human diseases [1,2,3,4], probably thanks to its antioxidant and anti-inflammatory activities. However, other resveratrol monomers are present in the human diet, such as piceatannol, pterostilbene, or astringin and piceid, as glycoside isoforms. Additionally, resveratrol oligomers consisting of 2–8 subunits of resveratrol could be present in grape [5]. One of these oligomers, ε-viniferin, is a resveratrol dimer and its concentration in wine is comprised between 0.1 and 4.3 mg/L [6]. ε-Viniferin has been shown, in vitro, to possess antioxidant [7], anti-inflammatory [8], anti-carcinogenic [9,10], and cardioprotective [11] activities.

In vivo, the bioavailability of resveratrol is reported to be low, notably because of its rapid and intensive conversion by the metabolism [12,13]. After absorption, resveratrol can be found in the blood stream in several forms such as the native form and as glucuronide or sulfate metabolites. Glucuronidation and sulfation, which can be performed by UDP-glucuronosyltransferase (UGT) and sulfotransferase (SULT), respectively, are major metabolic pathways for numerous polyphenols including resveratrol [14]. Both conjugations occur preferentially at the 3-OH group compared to the 4′-OH group [15,16]. In rats, the main metabolites are resveratrol-3-*O*-glucuronide and resveratrol-3-*O*-sulfate [17], whereas in human the 3-*O*-sulfate form is the major metabolite and the glucuronides are found only in small amounts [18].

Despite the low bioavailability of resveratrol and its derivates, their health benefits are well known. This discrepancy might be due to the biological activities of the metabolites themselves. For example, some metabolites, such as the sulfate derivatives of resveratrol, but not glucuronides, have been shown to inhibit the proliferation of human colorectal cancer cells [19], and to exert an anti-inflammatory effect [20]. In addition, sulfoconjugates are transported inside cells through the membrane transporter organic anion transporting polypetide [21] and glucuronides are excreted by members of the multridrug resistance-associated protein family [22]. Thus, the metabolism of stilbenes seems to play a crucial role in their disposition and their biological effects.

A recent study reported the extremely poor bioavailability of the resveratrol oligomers, but data about its metabolism are scarce and sometimes only partial [23,24,25]. In those papers, the identification of viniferin metabolites, i.e., sulfo- and glucuronoconjugates, was assumed to be indirect by observing an enhancement of the concentration of viniferin after enzymolysis by β-glucuronidase or sulfatase [24], or by the measurement of the consumption of viniferin after incubation with liver microsomes in the presence of uridine 5′-diphospho-glucuronic acid trisodium salt (UDPGA) [25]. Metabolism of ε-viniferin in rats and humans was not fully characterized, and we believe this is essential to decipher which entity is participating in the beneficial health effects. Therefore, this study was undertaken to characterize the metabolism of ε-viniferin and identify its metabolites in human and rat liver. First, all of the metabolites were obtained by chemical hemi-synthesis and were then identified in vitro in both species. In humans, ε-viniferin was intensively metabolized to glucurono- and sulfoconjugates to a similar degree, whereas glucuronoconjugates were mainly present in rats. We believe that the characterization of the metabolism of ε-viniferin will help to better understand its pharmacological activity in subsequent studies.

## 2. Results

### 2.1. Hemi-Synthesis and Structural Identification of ε-Viniferin Metabolites

**Figure 1:**
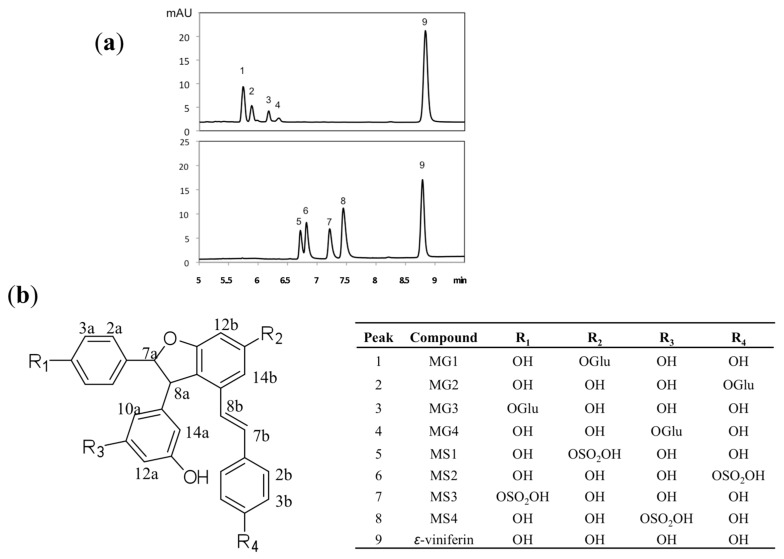
(**a**) UPLC-DAD profile of ε-viniferin and its eight metabolites (four glucuronides, upper panel and four sulfates, lower panel), produced by hemi-synthesis; (**b**) Structure of the eight metabolites. Glu = glucuronic acid.

First, the glucuronide and sulfate metabolites of ε-viniferin were hemi-synthesised according to the material and methods section. We chose this approach for several reasons. First, it allowed us to obtain all the complete metabolites likely to be produced in vivo and, second, it allowed us to obtain a sufficient quantity of metabolites for their identification at a lower cost than by using classical biosynthesis with S9 liver fractions. These metabolites were then purified and identified through mass spectrometry and NMR spectroscopy. Figure 1a shows the chromatograms of the eight hemi-synthesized metabolites. Mass spectra of 1, 2, 3, and 4 (MG1, MG2, MG3, and MG4, respectively) produced the quasi-molecular ion [M − H]^−^ at *m*/*z* 629, corresponding to the mass of ε-viniferin mono-glucuronide. The mass spectra of 5, 6, 7, and 8 (MS1, MS2, MS3, and MS4 respectively) produced the quasi-molecular ion [M − H]^−^ at *m*/*z* 533, corresponding to the ε-viniferin mono-sulfate.

Table 1 summarizes the NMR data of the eight ε-viniferin metabolites. Identification of glucuronide derivatives was based on NOESY correlations and chemical shift variations. Compound **1**, corresponding to MG1, shows NOEs between the anomeric proton (H-1′) and the H-12b and H-14b. In addition, the H-12b and H-14b protons were shifted upfield compared to that of ε-viniferin. These data indicate that glucuronic acid is attached to the C-13b carbon. Concerning 2 (MG2), NOE correlation between the anomeric proton and H-3b/5b protons indicated that glucuronic acid is attached to the C-4b carbon. This hypothesis was confirmed by the chemical shift variations of H-2a/6a and H-3b/5b protons. The NOESY spectrum of 3 (MG3) showed a correlation between the anomeric proton and H-3a/5a, indicating the position of glucuronic acid. Finally, the NOEs between H-1′ and H-10a/H-12a protons and the chemical shift variations allowed the identification of 4 (MG4). Like the glucuronide derivatives, the sulfate metabolites 5, 6, 7, and 8 (MS1, MS2, MS3, and MS4, respectively) were based on chemical shift variations of ε-viniferin. The chemical structures of ε-viniferin and its metabolites are shown in Figure 1b.

### 2.2. In Vitro ε-Viniferin Conjugates Profiling in Humans and Rats

**Figure 2:**
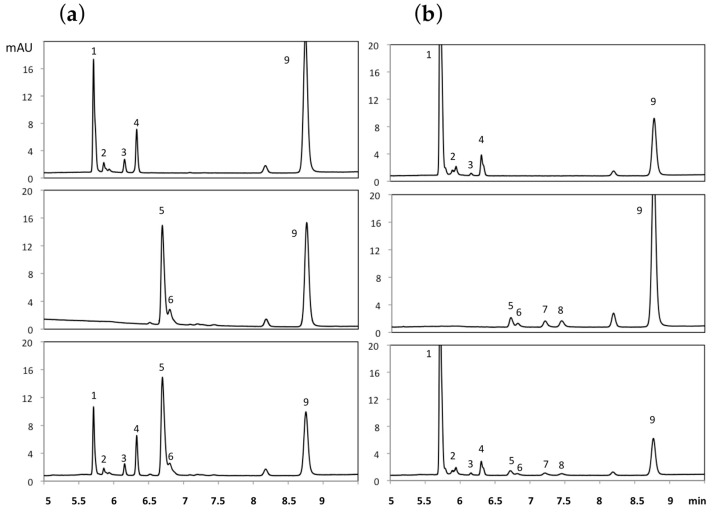
UPLC-DAD profile of ε-viniferin and its metabolites. ε-viniferin (50 µM) was incubated with 2 mg/mL protein S9 fraction from human (**a**) or rat (**b**) liver at 37 °C for 40 min. The co-factors used were UDPGA (**upper panel**), PAPS (**middle panel**), or the combination of the two (**lower panel**). 1 = MG1, 2 = MG2, 3 = MG3, 4 = MG4, 5 = MS1, 6 = MS2, 7 = MS3, 8 = MS4, and 9 = ε-viniferin.

After incubation of ε-viniferin (50 µM) for 40 min at 37 °C with either human (Figure 2a, lower panel) or rat (Figure 2b, lower panel) liver S9 fractions (2 mg/mL) in the presence of different phase II cofactor(s), i.e., 3′-phosphoadenosine-5′-phosphosulfate (PAPS) and UDPGA, eight metabolites were found using UPLC-DAD-MS. These metabolites were further identified by comparison with the UPLC retention times and mass spectra of the authentic chemical standards that were obtained by chemical hemi-synthesis (Figure 1a). These eight compounds were the four glucuronoconjugates (MG1, MG2, MG3, and MG4) and the four sulfoconjugates (MS1, MS2, MS3, and MS4). These metabolites were absent in the control condition, i.e., in the presence of liver S9 fractions and in the absence of phase II cofactors (data not shown).

In humans, incubation of ε-viniferin with UDPGA showed the presence of four mono-glucuronides, MG1, MG2, MG3, and MG4. Figure 2a (upper panel) also showed that MG1 and MG4 were the major metabolites among the glucuronides. Incubation of ε-viniferin with PAPS revealed the presence of two major mono-sulfates, namely MS1 and MS2 (Figure 2a, middle panel). MS1 was the major metabolite among the sulfoconjugates. MS3 and MS4 were observed only in trace amount, so they were not studied further.

In rat, incubation of ε-viniferin with UDPGA revealed the presence of four mono-glucuronides, namely MG1, MG2, MG3 and MG4 (Figure 2b, upper panel). MG1 was the major metabolite among the glucuronides. Incubation of ε-viniferin with PAPS revealed the presence of four mono-sulfoconjugates, MS1, MS2, MS3, and MS4, which were present in almost equal amounts (Figure 2b, middle panel).

### 2.3. Kinetic Studies for Phase II Metabolism of ε-Viniferin

To further understand the contribution of glucuronidation or sulfation metabolic pathways to ε-viniferin in vitro clearance, kinetic studies were performed using the liver S9 fraction. The kinetic parameters are summarized in Table 2 for glucuronidation and sulfation. In rat, of the two conjugation pathways, the V_max_/K_m_ value for glucuronidation was higher than sulfation (20.08 µL/min/mg protein vs. 2.59 µL/min/mg protein, which represents the sum of the individual V_max_/K_m_ values for glucuronidation and sulfation, respectively). In humans, however, the V_max_/K_m_ values for glucuronidation and sulfation were quite similar (4.98 µL/min/mg protein vs. 6.35 µL/min/mg protein, which represents the sum of the individual V_max_/K_m_ values for glucuronidation and sulfation, respectively).

**Figure 3:**
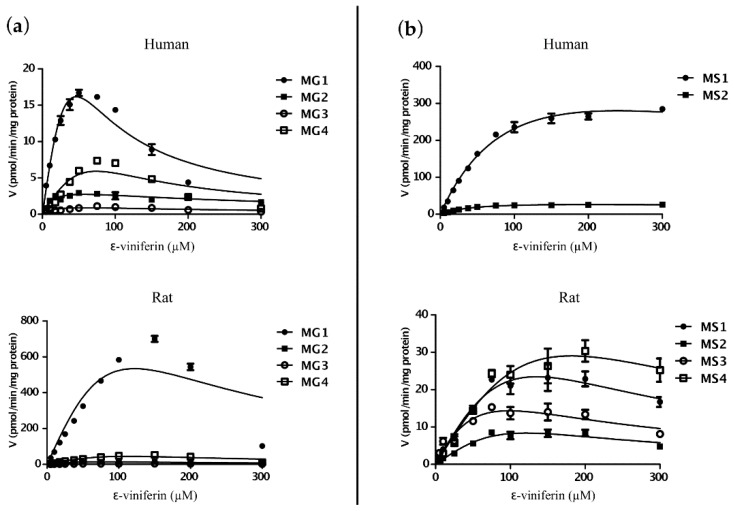
Enzyme kinetics of glucuronidation (**a**) and sulfation (**b**) of ε-viniferin by human and rat liver S9 fractions. Experimental procedures are described under Materials and Methods section. Data are represented by the mean ± SEM of three independent experiments.

For both species, glucuronidation and sulfation of ε-viniferin corresponded best to the substrate-inhibition model (Figure 3). The maximal velocity rate (V_max_) for the glucuronides was observed with MG1 in both species (Table 2a) while the maximal velocity rate for the sulfates was observed with MS1 in human and MS4 in rat (Table 2b).

### 2.4. Interspecies Differences in Phase II Metabolism of ε-Viniferin

The data collected for the glucuronidation and sulfation of ε-viniferin in both species enabled us to compare the phase II metabolism of ε-viniferin by humans vs. rats and to determine the interspecies differences. Table 3 shows the ratio of formation of ε-viniferin glucuronides and sulfates after 40 min incubation with liver S9 fractions. The formation of phase II metabolites occurred in both species but with some differences. Biotransformation of ε-viniferin was estimated to be around 72.7% and 78.1% in human and rat liver S9 fractions, respectively. In rats, sulfation was responsible for 4.3% of the metabolism of ε-viniferin, whereas in humans it was responsible for 43.6%. In contrast, glucuronidation of ε-viniferin occurred for 73.8% in rat and 29.1% in human. MG1 was the main glucuronide observed in rats, whereas the main ones in humans were MG1 and MG4. Four of the sulfates were observed in rats in equal amounts, but only two in humans, MS1 and MS2.

## 3. Discussion

It has been recently shown that ε-viniferin and δ-viniferin have a low bioavailability in mice and rats, respectively, after oral administration [23,24,26]. The poor bioavailability of δ-viniferin is thought to be due to its intense metabolism to glucurono- and sulfoconjugates [24]. Nevertheless, the metabolites of these two resveratrol dimers have not been characterized, although this is an essential step for deciphering which entity plays a role in these beneficial health effects.

Therefore, this study was undertaken to identify and characterize the metabolites of ε-viniferin and to describe the kinetic profile of their appearance in human and rat. The rat model was chosen as a prerequisite for further studies that to be performed in vivo. In addition, it allowed us to compare the metabolite profile between these species and to investigate whether the rat could be a suitable model for extrapolating the metabolism of ε-viniferin in human.

The study sought to establish the glucuronidation and sulfation processes involved. The biotransformation of polyphenols is a complex process and some authors have reported the formation of various disulfate and diglucuronide forms of resveratrol [18,27]. In our study, we did not observe these metabolites.

After incubation of ε-viniferin with liver S9 fractions, we identified eight metabolites, four glucurono- and four sulfo-conjugates. In both species, ε-viniferin was subjected to an intense metabolism as approximately 70–80% of the molecules were converted to conjugates. These results are in accordance with data showing that glucuronidation and sulfation are the main biotransformation pathways for stilbenes such as resveratrol and piceatannol [13,28,29]. Nevertheless, our findings highlight some differences between the profiles of human and rat metabolites.

In rat, glucuronidation is the main metabolic pathway as more than 73% of ε-viniferin is biotransformed through this reaction. Among the glucuronides, MG1 is the major metabolite as shown in Figure 2. For sulfation we observed four sulfoconjugates in rat. Of the two metabolic pathways, glucuronidation might be the main process for the hepatic clearance of ε-viniferin, as shown by the high V_max_/K_m_ values as compared to sulfation (Table 2). Mao et al. [24] investigated oral or intravenous administration of another dimer of resveratrol, δ-viniferin, in rats. They also reported that glucuronides were the main compounds.

In humans, we could only quantify two sulfoconjugates. However, we noticed that sulfation was more involved in the biotransformation of ε-viniferin than glucuronidation in vitro (43.6% vs. 29.1%, Table 3). Nevertheless, enzyme activities could be influenced by hepatic cofactor concentrations (PAPS and UDPGA) different in vivo that those used in vitro. Our results suggest that both pathways, glucuronidation and sulfation, could be involved in the hepatic clearance of this resveratrol dimer in humans.

Therefore, there are some differences in the metabolism of ε-viniferin between species, with glucuronidation predominating in rats and both glucuronidation and sulfation in humans. Previous studies have shown that glucuronidation might be the major metabolic pathway for some polyphenols in various species. For resveratrol and ε-viniferin, it was shown that rodent microsomes, i.e., rat and mice, have a significantly higher activity for glucuronidation than in human [25,30]. Our data show that this glucuronidation process also seems to be more effective in rat than in human in S9 fractions. By contrast, for sulfation pathway, it seemed that the differences in the metabolic profile for ε-viniferin are different to those observed for resveratrol. For example, resveratrol sulfate was found to be more abundant in rat hepatocytes than in human hepatocytes [31].

Among the glucuronides, the main metabolites, MG1 and MG4, were generated in an average ratio (MG4:MG1) of 1:15 in rats and 1:1.5 in humans. This interspecies difference was already observed in the formation of resveratrol glucuronides. Indeed, it has been shown that resveratrol can be metabolized by human and rat liver microsomes into resveratrol-3-*O*-glucuronide and resveratrol-4′-*O*-glucuronide with a preference for the 3-position, especially in rats [15,30]. We found that glucuronidation occurs at positions R2 and R3 of viniferin (MG1 and MG4, respectively), which is also consistent with the pattern of glucuronidation in the 3-position of resveratrol.

Recent studies reported that the oral bioavailability of δ-viniferin and ε-viniferin are 2.3% and 0.77%, respectively [23,24]. Thus, the oral bioavailability of these two dimers of resveratrol is extremely low, perhaps owing to their low absorption through the intestinal epithelium and to their intense metabolism. We have already demonstrated that ε-viniferin can undergo an intense metabolism by the hepatic tissue. The metabolism of viniferin by the microbiote or by the intestinal tissue has been little investigated. Indeed Willenberg et al. shown a low intestinal absorption and conjugation rate of ε-viniferin in vitro [32]. Thus, as we have now identified glucurono- and sulfoconjugates produced by human and rat liver S9 fractions, we believe that further studies of ε-viniferin metabolism by the intestinal tissues are needed in order to better understand its pharmacokinetic in vivo. These pharmacokinetic studies are of great interest and are currently in progress in rats in our laboratory, in order to identify the metabolic profile in vivo and further characterize the active metabolites of viniferin.

ε-Viniferin is known to have a variety of pharmacological effects, such as anti-inflammatory [33], anti-oxidant [7], hepatoprotective [34], antiproliferative [35], and cardioprotective [36] ones. For some activities, such as cardioprotection, the effect of ε-viniferin has been reported to be greater than that elicited by its monomer resveratrol [11]. By contrast, for some antiproliferative activities in human leukemia or colonic cancer cells lines, ε-viniferin was found to be less effective than the monomer [9,10]. These differences might depend on the models used but also of the different metabolites formed in them.

One could question whether the native form of viniferin or its metabolites could have pharmacological properties given its extremely low bioavailability. The beneficial health effects of polyphenols have been demonstrated for several years both in human and animal studies. The discrepancy between the beneficial health effects and the poor bioavailability due to extensive biotransformation may be accounted for the pharmacological action of the metabolites. For resveratrol, there is evidence that its conjugates could elicit biological activities. Indeed, sulfoconjugates can induce quinone reductase, inhibit cyclooxygenase [37,38] and act as an anti-inflammatory agent [20] while glucuronides have a stronger antioxidant activity than the unconjugated form [39]. In addition it was demonstrated that exposure to sulfoconjugates of resveratrol could induce autophagy and senescence in cancer cells, and this effect was inhibited by sulfatases inhibitors. Those results suggested that sulfoconjugates could penetrate inside cancer cells where they have liberated free resveratrol after hydrolysis by sulfatases, therefore highlighting the role of metabolism in the disposition of the active parent compound [40]. Data on the biological activities of the metabolites of resveratrol oligomers are scarce. For this reason, our laboratory is now producing a large amount of ε-viniferin metabolites in order to test their biological activities.

## 4. Materials and Methods

### 4.1. Materials

ε-Viniferin was produced and purified in our laboratory (Unité de recherché Œnologie—EA 4577, Villenave d’Ornon, France). Pyridine, chlorosulfonic acid, acetobromo-glucuronic acid methyl-ester, sodium hydroxide (NaOH), uridine 5′-diphospho-glucuronic acid trisodium salt (UDPGA), alamethicin, 3′-phosphoadenosine-5′-phosphosulfate (PAPS), dithiothreitol (DTT), trifluoroacetic acid (TFA), formic acid (FA), and MgCl_2_ were purchased from Sigma-Aldrich (Saint-Quentin Fallavier, France). Human liver S9 and rat liver S9 fractions were purchased from Biopredic International (Saint-Grégoire, France). Acetonitrile and methanol were purchased from Thermo Fisher Scientific (Illkirch, France).

### 4.2. Hemi-Synthesis of ε-Viniferin Sulfates

To a chilled (−5 °C) solution of ε-viniferin (50 mg, 0.11 mmol) in 5 mL of pyridine, chlorosulfonic acid (0.3 mL, 1.1 mmol) was added dropwise. When the addition was complete, the reaction was placed at room temperature for 1 h. Then acidified water (0.5% FA) was added in order to reach a pH of 3 and the mixture was evaporated. The solid obtained was then re-dissolved in water (2 mL) prior to purification by semi-preparative HPLC-DAD to furnish after freeze-drying MS1 (3.64 mg, 6.2%), MS2 (5.05 mg, 8.6%), MS3 (5.40 mg, 9.2%), and MS4 (9.40 mg, 16%).

### 4.3. Hemi-Synthesis of ε-Viniferin Glucuronides

Monoglucuronides of ε-viniferin were obtained by chemical *O*-glucuronidation of the previously purified ε-viniferin using acetobromo-glucuronic acid methyl ester in alkaline conditions. ε-Viniferin (50 mg, 0.11 mmol) was dissolved in dry ethanol (5 mL) and NaOH (26 mg, 0.66 mmol) was added. The solution was then placed at 50 °C for 30 min before the addition of acetobromo-glucuronic acid methyl ester (87 mg, 0.22 mmol) also dissolved in dry ethanol (5 mL). The reaction mixture was kept at 50 °C and in the dark for 4 h. Then after cooling down, acidified water (0.5% FA) was added in order to reach a pH of 3 and the mixture was evaporated. The solid obtained was then re-dissolved in water (2 mL) prior to purification by semi-preparative HPLC-DAD to furnish after freeze-drying MG1 (6.10 mg, 8.8%), MG2 (3.33 mg, 4.8%), MG3 (2.91 mg, 4.2%), and MG4 (1.52 mg, 2.2%).

### 4.4. Semi-Preparative HPLC of Metabolites

Samples from chemical hemi-synthesis were separated by HPLC equipped with a binary pump, an UV-VIS detector (Prostar 325, Varian, Palo Alto, CA, USA), and Prontosil C18 column (5 µm, 8 × 250 mm). Detection was carried out at 320 nm.

Solvents and the gradient employed for the separation of ε-viniferin metabolites were as follows: solvent A (H_2_O 0.025% TFA); solvent B (acetonitrile 0.025% TFA); gradient program 0–4 min, 10% B; 4–9 min, 10–20% B; 9–13 min, 20–30% B; 13–17 min, 30% B; 17–21 min, 30–35%; 21–30 min, 35–60%; 30–38 min, 60–100%; 38–44 min, 100% (Figure S1, Supplementary Materials). The flow rate was set to 3 mL/min. Peaks were collected, concentrated under vacuum, and freeze-dried. The metabolites were identified by HPLC-DAD-MS and NMR analysis.

### 4.5. UPLC-DAD-MS Analysis

Samples from either chemical hemi-synthesis or metabolic reactions were carried using a 1290 Infinity UPLC (Agilent Technologies, Courtaboeuf, France). The UPLC was coupled to an Esquire 3000 Plus ion trap mass spectrometer using an ESI source (Bruker-Daltonics, Billerica, MA, USA). 2 µL were injected into an Agilent SB-C18 column (1.8 µm, 2.1 × 100 mm). Samples were eluted with solvent A (H_2_O 0.1% FA) and solvent B (acetonitrile 0.1% FA) by the following gradient program: 0–1.7 min, 10% B; 1.7–3.4 min, 10–20% B; 3.4–5.1 min, 20–30% B; 5.1–7.8 min, 30% B; 7.8–8.5 min, 30–35% B; 8.5–11.9 min, 35–60% B; 11.9–15.3 min, 60–100% B; 15.3–17 min, 100% B; 17–17.3 min, 100–10% B. The flow rate was set to 0.4 mL/min and the UV detector was set at the wavelength 320 nm. Total ion chromatograms were obtained using negative mode with a range of *m*/*z* 130–1400. The parameters were: capillary voltage, +4 kV; nebulizer pressure, 40 psi; dry gas, 10 L/min; dry temperature, 365 °C. Data analysis was performed with Bruker Data Analysis 3.2 (Bruker-Daltonics, Billerica, MA, USA). Metabolite concentrations were expressed as an equivalent of the ε-viniferin standard curve.

### 4.6. NMR Analysis

NMR experiments were performed on a Bruker Avance III 600 MHz (Bruker-Daltonics, Billerica, MA, USA) spectrometer equipped with a 5-mm triple-resonance probe. Chemical shifts were calibrated using residual solvent signal (acetone-*d*_6_) at 2.05 ppm. Assignments were performed with COSY and ROESY experiments.

### 4.7. Kinetic Study

The incubation conditions were optimized by preliminary experiments to ensure the formation rates of metabolites were linear over the incubation time and at the concentration of protein added. Sulfation was investigated by incubating human or rat liver S9 fractions (2 mg/mL) with ε-viniferin at different concentrations (5 to 300 µM) in Tris-HCl buffer (50 mM, pH 7.4), in the presence of PAPS (1 mM), DTT (10 mM), MgCl_2_ (5 mM) in a final volume of 100 µL. After 40 min at 37 °C, the reaction was stopped with 100 µL of methanol to precipitate the protein. Glucuronidation was investigated by incubating human or rat liver S9 fractions (2 mg/mL) with ε-viniferin at different concentrations (5 to 300 µM) in Tris-HCl buffer (50 mM, pH 7.4), in the presence of UDPGA (1 mM), alamethicin (25 µg/mL), and MgCl_2_ (5 mM) in a final volume of 100 µL. After 40 min at 37 °C, the reaction was stopped with 100 µL of methanol to precipitate the protein. Samples from sulfation and glucuronidation were then centrifuged for 30 min (14,000× *g*, 4 °C), and the resultant supernatants were analyzed using UPLC-DAD-MS as described above. The kinetic model that was used to analyze the results was substrate inhibition. Kinetic parameters, i.e., V_max_ and K_m_ were obtained using GraphPad Prism software (La Jolla, CA, USA). Results are expressed as mean ± sem of three independent experiments.


V = (V_max_ × [S])/(K_m_ + [S] + [S]^2^/K_i_),

V_max_: maximal velocity; K_m_: substrate affinity constant; [S]: substrate concentration; K_i_: substrate inhibition constant.

## 5. Conclusions

In conclusion, we provide evidence of a strong in vitro hepatic clearance of ε-viniferin and have characterized its metabolite profile in humans and rats. Glucuronidation and sulfation are both involved in humans to a similar degree whereas glucuronidation is the main pathway in rats. This suggests that rats are not the best animal model to study ε-viniferin metabolism in humans. Nevertheless, pharmacokinetic studies play a pivotal role in drug development and rat studies are essential. The beneficial health effects of polyphenols in vivo are now well known despite their low bioavailability, which might be partly due to their intense metabolism. Therefore, our findings underline the need to characterize the active metabolites of ε-viniferin, because this might explain differences in its beneficial health effects between species according to its metabolism.

## Figures and Tables

**Table 1:** ^1^H-NMR data for ε-viniferin and its metabolites.

n°	ε-Viniferin	1	2	3	4	5	6	7	8
2a/6a	7.17 d(8)	7.17 d(8)	7.18 d(8)	7.26 d(8)	7.18 d(8)	7.17 d(8)	7.17 d(8)	7.17 brs	7.17 d(8)
3a/5a	6.73 d(8)	6.74 d(8)	6.74 d(8)	6.98 d(8)	6.77 d(8)	6.73 d(8)	6.73 d(8)	7.17 brs	6.73 d(8)
7a	5.42 d(5)	5.45 d(5)	5.46 d(5)	5.42 d(5)	5.47 d(5)	5.44 d(5)	5.46 d(5)	5.46 d(5)	5.46 d(5)
8a	4.47 d(5)	4.52 d(5)	4.48 d(5)	4.49 d(5)	4.54 d(5)	4.51 d(5)	4.51 d(5)	4.48 d(5)	4.50 d(5)
10a	6.24 brs	6.23 brs	6.25 d(2)	6.24 brs	6.59 brs	6.25 brs	6.26 d(2)	6.26 brs	6.69 t(2) ^(1)^
12a	6.24 brs	6.23 brs	6.24 t(2)	6.24 brs	6.50 brs	6.25 brs	6.24 t(2)	6.26 brs	6.32 t(2) ^(1)^
14a	6.24 brs	6.23 brs	6.25 d(2)	6.24 Brs	6.35 brs	6.25 brs	6.26 d(2)	6.26 brs	6.89 t(2) ^(1)^
2b/6b	7.20 d(8)	7.20 d(8)	7.30 d(8)	7.21 d(8)	7.21 d(8)	7.21 d(8)	7.29 d(8)	7.21 d(8)	7.21 d(8)
3b/5b	6.83 d(8)	6.83 d(8)	7.09 d(8)	6.83 d(8)	6.84 d(8)	6.83 d(8)	7.24 d(8)	6.83 d(8)	6.83 d(8)
7b	6.90 d(16)	7.00 d(16)	6.90 d(16)	6.94 d(16)	6.91 d(16)	6.93 d(16)	6.88 d(16)	6.91 d(16)	6.88 d(16)
8b	6.71 d(16)	6.73 d(16)	6.71 d(16)	6.78 d(16)	6.71 d(16)	6.71 d(16)	6.71 d(16)	6.77 d(16)	6.70 d(16)
12b	6.32 d(2)	6.58 d(2)	6.34 d(2)	6.34 d(2)	6.35 d(2)	6.87 brs	6.34 d(2)	6.34 d(2)	6.33 d(2)
14b	6.72 d(2)	6.99 d(2)	6.72 d(2)	6.73 d(2)	6.72 d(2)	7.11 brs	6.71 d(2)	6.73 d(2)	6.71 d(2)
1′		5.18 d(7)	5.13 d(7)	5.09 d(7)	5.10 d(7)				
2′		3.56 t(8)	3.53 t(8)	3.51 t(8)	3.51 t(8)				
3′		3.64 t(9)	3.59 t(9)	3.58 t(9)	3.60 t(9)				
4′		3.73 t(9)	3.69 t(9)	3.70 t(10)	3.72 t(9)				
5′		4.18 d(10)	4.11 d(10)	4.11 d(10)	4.10 d(10)				

^(1)^ Ambiguous assignments.

**Table 2:** Kinetic parameters of ε-viniferin glucuronidation (**a**) and sulfation (**b**) by human or rat liver S9 fractions.

**(a)**	**Species**	**Glucuronides**	**K_m_**	**V_max_**	**V_max_/K_m_**		
	Human	MG1	3.03 ± 3.00	11.27 ± 1.30	3.72	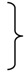	4.98
		MG2	4.20 ± 1.88	2.54 ± 0.16	0.60		
		MG3	2.78 ± 2.00	0.78 ± 0.06	0.28		
		MG4	13.18 ± 9.39	4.91 ± 0.78	0.37		
	Rat	MG1	32.94 ± 16.94	548.90 ± 86.17	16.66	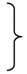	20.08
		MG2	9.09 ± 4.26	12.49 ± 1.13	1.37		
		MG3	8.52 ± 2.10	3.06 ± 0.14	0.36		
		MG4	26.14 ± 11.55	43.99 ± 5.49	1.68		
**(b)**	**Species**	**Sulfates**	**K_m_**	**V_max_**	**V_max_/K_m_**		
	Human	MS1	66.67 ± 6.20	361.60 ± 13.09	5.42	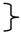	6.35
		MS2	33.01 ± 3.43	30.46 ± 0.96	0.92		
	Rat	MS1	34.93 ± 11.33	25.16 ± 2.29	0.72	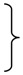	2.59
		MS2	27.24 ± 11.15	8.46 ± 0.89	0.31		
		MS3	13.23 ± 5.28	13.47 ± 1.10	1.02		
		MS4	68.60 ± 20.35	36.79 ± 3.94	0.54		

Units: K_m_, µM; V_max_, pmol/min/mg protein; V_max_/K_m_, µL/min/mg protein. Data are represented by the mean ± SEM of three independent experiments.

**Table 3:** Interspecies differences in the formation of glucurono- and sulfoconjugates of ε-viniferin (50 µM) incubated during 40 min in the presence of liver S9 fractions.

	Human Liver		Rat Liver	
ε-viniferin conversion (%)	72.7 ± 0.7		78.1 ± 0.9	
Glucuronidation (%)	29.1 ± 1.2	*MG1: 54.6 ± 2.4*	73.8 ± 1.5	*MG1: 91.1 ± 2.4*
		*MG2: 5.0 ± 1.2*		*MG2: 1.1 ± 0.2*
		*MG3: 8.9 ± 0.5*		*MG3: 0.7 ± 0.0*
		*MG4: 31.5 ± 0.9*		*MG4: 7.1 ± 0.1*
Sulfatation (%)	43.6 ± 1.8	*MS1: 75.2 ± 3.8*	4.3 ± 0.3	*MS1: 38.0 ± 1.4*
		*MS2: 24.8 ± 1.2*		*MS2: 23.5 ± 3.1*
		*MS3: 0*		*MS3: 22.0 ± 1.9*
		*MS4: 0*		*MS4: 16.4 ± 1.3*

In italics, the percentage of each metabolite is expressed relative to the total amount of ε-viniferin metabolites formed for the respective metabolic reaction.

## Supplementary Materials

Supplementary materials are available on line: Figure S1. Semi-preparative HPLC profile of ε-viniferin and its metabolites (four glucuronides, upper panel and four sulfates, lower panel), produced by hemi-synthesis.
