# Supplementary material for: In Vitro Glucuronidation and Sulfation of ε-Viniferin, a Resveratrol Dimer, in Humans and Rats
Source: Molecules. 2017 May 3;22(5):733. doi: 10.3390/molecules22050733 (PMC6154661; doi:10.3390/molecules22050733)
Supplement: Supplementary file 1 [file molecules-22-00733-s001.pdf]

Supplementary Material: In Vitro Glucuronidation and Sulfation of ε-Viniferin, A Resveratrol Dimer, in Human and Rat

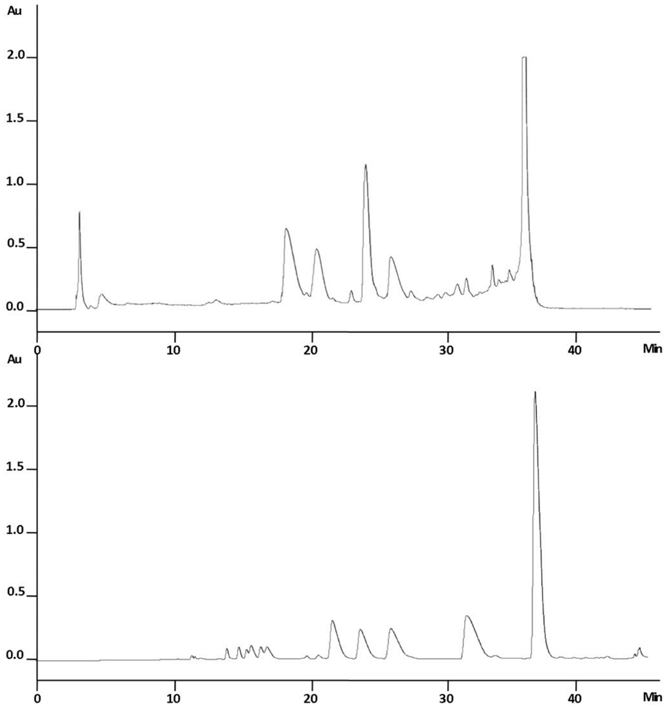

Figure S1. Semi-preparative HPLC profile of *ε*-viniferin and its metabolites (four glucuronides, upper panel and four sulfates, lower panel), produced by hemi-synthesis.
